# Functional Diversities of Regulatory T Cells in the Context of Cancer Immunotherapy

**DOI:** 10.3389/fimmu.2022.833667

**Published:** 2022-03-17

**Authors:** Ran Gao, Guo-Ping Shi, Jing Wang

**Affiliations:** ^1^ State Key Laboratory of Medical Molecular Biology, Institute of Basic Medical Sciences, Chinese Academy of Medical Sciences, Department of Pathophysiology, Peking Union Medical College, Beijing, China; ^2^ Department of Medicine, Brigham and Women’s Hospital, Harvard Medical School, Boston, MA, United States

**Keywords:** regulatory T cell, tumor, immunosuppression, immunotherapy, radiotherapy

## Abstract

Regulatory T cells (Tregs) are a subset of CD4^+^ T cells with their immunosuppressive activities to block abnormal or excessive immune responses to self and non-autoantigens. Tregs express the transcription factor Foxp3, maintain the immune homeostasis, and prevent the initiation of anti-tumor immune effects in various ways as their mechanisms to modulate tumor development. Recognition of different phenotypes and functions of intratumoral Tregs has offered the possibilities to develop therapeutic strategies by selectively targeting Tregs in cancers with the aim of alleviating their immunosuppressive activities from anti-tumor immune responses. Several Treg-based immunotherapeutic approaches have emerged to target cytotoxic T lymphocyte antigen-4, glucocorticoid-induced tumor necrosis factor receptor, CD25, indoleamine-2, 3-dioxygenase-1, and cytokines. These immunotherapies have yielded encouraging outcomes from preclinical studies and early-phase clinical trials. Further, dual therapy or combined therapy has been approved to be better choices than single immunotherapy, radiotherapy, or chemotherapy. In this short review article, we discuss our current understanding of the immunologic characteristics of Tregs, including Treg differentiation, development, therapeutic efficacy, and future potential of Treg-related therapies among the general cancer therapy.

## Introduction

Thymic T cells express T-cell receptor (TCR) at the mature stage when thymus produces immune functional T cells through positive and negative selection to recognize endogenous or exogenous antigens. The purpose of positive selection is to make those T cells carrying TCR that receives survival signals from the major histocompatibility complex-II (MHC-II) molecules on thymic cells, while negative selection mainly leads to the exclusion of T cells with high affinity for MHC-bound antigen peptides. However, some T cells with self-reactive TCR can develop into CD4^+^CD25^+^Foxp3^+^ regulatory T cells (Tregs) after interacting with autoantigen peptides with high affinity (1). Tregs are an immunosuppressive subgroup of the CD4^+^ T cells. The concept of immunosuppressive T cells was proposed in 1970s (2, 3). A decade later, reports confirmed that Tregs exerted their immunosuppressive effects in mouse tumor models (4, 5). Such immunosuppressive effect of Tregs was further confirmed in 1995 (6). At present, many different types of Tregs have been identified. Naturally developed Tregs account for 5% to 10% of total peripheral CD4^+^ T cells and are characterized by their high expression of CD25 and low expression of CD45RB (7–9). While the IL-2 receptor CD25 serves as a surface marker of suppressor T cells (10), the forkhead/winged helix transcription factor (Foxp3) is considered a classical combined marker of Tregs (11). The most prominent Treg types include thymus-derived Tregs (tTregs), peripherally generated Tregs (pTregs) from the Foxp3^–^ T conventional (Tconv) cells, and *in vitro* interleukin-2 (IL-2) and transform growth factor-β (TGF-β)-induced Tregs (iTregs) from Tconv cells (12). tTregs are generated in the thymus through MHC class II-dependent TCR interactions that result in high-avidity selection. Activated polyclonal tTregs modulate T-effector cell trafficking to the target organs, while antigen-specific iTregs inhibit T-effector cell priming by targeting the antigen presenting cells (APCs) (13).

Both tTregs and pTregs are stable in the expression of *Foxp3* and other Treg signature genes such as *Cd25* and cytotoxic T lymphocyte antigen-4 (*Ctla4*). These Tregs show sustained immunosuppressive function. The intronic enhancers CNSs (conserved non-coding sequences), also known as TSDRs (Treg-specific demethylation regions), and promoter of the *Foxp3* gene play important role in *Foxp3* gene stable expression (14, 15). In contrast, the expression of *Foxp3* and Treg signature genes in iTregs remains unstable due to incomplete epigenic changes at the TSDRs and these iTregs may become T-effector cells under certain *in vivo* conditions (16). The stability of Foxp3 expression and immunosuppressive functionality of iTregs relies on the efficient demethylation of the CpG island in the first intron of *Foxp3* gene locus CNS2 region (16–19). CNS2 demethylation enhances the recruitment of transcription factors STAT5 (signal transducer and activator of transcription 5), NFAT (nuclear factor of activated T cells), Runx1/Cbfβ, CREB (cAMP-response element binding protein), and Foxp3 itself (20, 21). While reduced demethylation of CNS2 in iTregs leads to impaired Foxp3 expression and iTreg function stability (22), complete demethylation of CNS2 is required for optimal *Foxp3* gene expression and iTreg immunosuppression activity (23). In addition to CNS2 demethylation, other key factors determining the development of iTregs include the types of APCs, their differentiation status, and cytokine environment in the activation process.

Tumor infiltrating dendritic cells (DCs), and TGF-β, IL-2, and indoleamine-2, 3-dioxygenase-1 (IDO-1) are all essential cells and molecules that promote CD4^+^ T-cell differentiation into Tregs (24, 25). Tregs have been among the most extensively studied lymphocytes in oncology for decades. Yet, the successful and precise targeting of Tregs for cancer immunotherapy has been elusive, although these cells may exert different functions depending on their residential tissue types. For example, multiple classes of genes are differentially regulated in Tregs from the visceral adipose tissue (VAT) compared with those in the lymphoid organs, including those encoding the transcription factors, chemokines and cytokines and their receptors, and molecules that are implicated in lipid metabolism to regulate adipose tissue homeostasis and organismal metabolism. These Tregs display much more restricted repertoire of antigen-specific TCRs and stronger dependency on the cytokine IL-33 and its receptors ST2 than those in the lymphoid organs (26, 27). Skeletal muscle Tregs are expanded in response to acute or chronic injury. Like the VAT Tregs, skeletal muscle Tregs also express high levels of transcription factors, chemokines, cytokines, and their receptors (26). The colonic Tregs are developed against microbial antigens. Mice devoid of any microbiota showed much smaller number of colonic Tregs than those in specific pathogen-free (SPF) mice (28). Intestinal Tregs express high levels of ST2 and tissue repair factors. These cells also express inducible costimulator (ICOS), CTLA-4, and ectonucleotidases CD39 and CD73 to regulate Th2- and Th1/Th17-mediated immunity (29). Tregs in the skin are involved in regulating microbial colonization, wound healing, and hair follicle development (29). In tumor microenvironment, Tregs inhibit the antitumor immunity and promote tumor occurrence and development by suppressing the function of immune effector cells *via* a variety of mechanisms (30) that will be discussed here. Emerging evidence suggests that Tregs demonstrate remarkable adaptability to their local environment and facilitate the immune homeostasis through highly specialized tissue-specific pathways (31). After effective elimination of pathogenic threats, the evolutionarily evolved immune system immediately restores the quiescence and prevents further harm (32).

## Regulatory T-Cell Immunosuppressive Function Regulation

Tregs are important mediators of the peripheral tolerance to autoantigens and non-autoantigens, which can be controlled by a variety of inhibitory mechanisms. Treg differentiation, proliferation, and immunosuppression activity vary in response to environmental signals that may alter Treg stability, plasticity, and tissue-specific heterogeneity and shape Treg environmental-dependent immunosuppressive functions (10, 33, 34). These signals include cell-extrinsic factors, such as nutrients, vitamins, and metabolites, and cell-intrinsic metabolic programs. Foxp3 is the major regulatory molecule of Tregs that produces and maintains Treg immunosuppressive activity. Studies have found that lack of Foxp3 in mice increased T-effector cell activity due to Treg depletion (35). Like Treg depletion, mutations of Foxp3 caused fatal immune dysregulation-associated multi-organ autoimmune diseases, such as polyendocrinopathy, enteropathy, and X-linked syndrome (36–38). Transfer of donor CD4^+^CD25^+^ Tregs into neonatal Foxp3-deficient mice rescued the lymphoproliferative disorder in recipient mice, suggesting that Foxp3 is a critical regulator of CD4^+^CD25^+^ Treg function (11). Yet, both extrinsic and intrinsic factors may enhance or impair Treg differentiation, proliferation, and immunosuppression function, depending on the Treg residential environment and their immune status.

### Treg Surface Molecules

**Figure 1:**
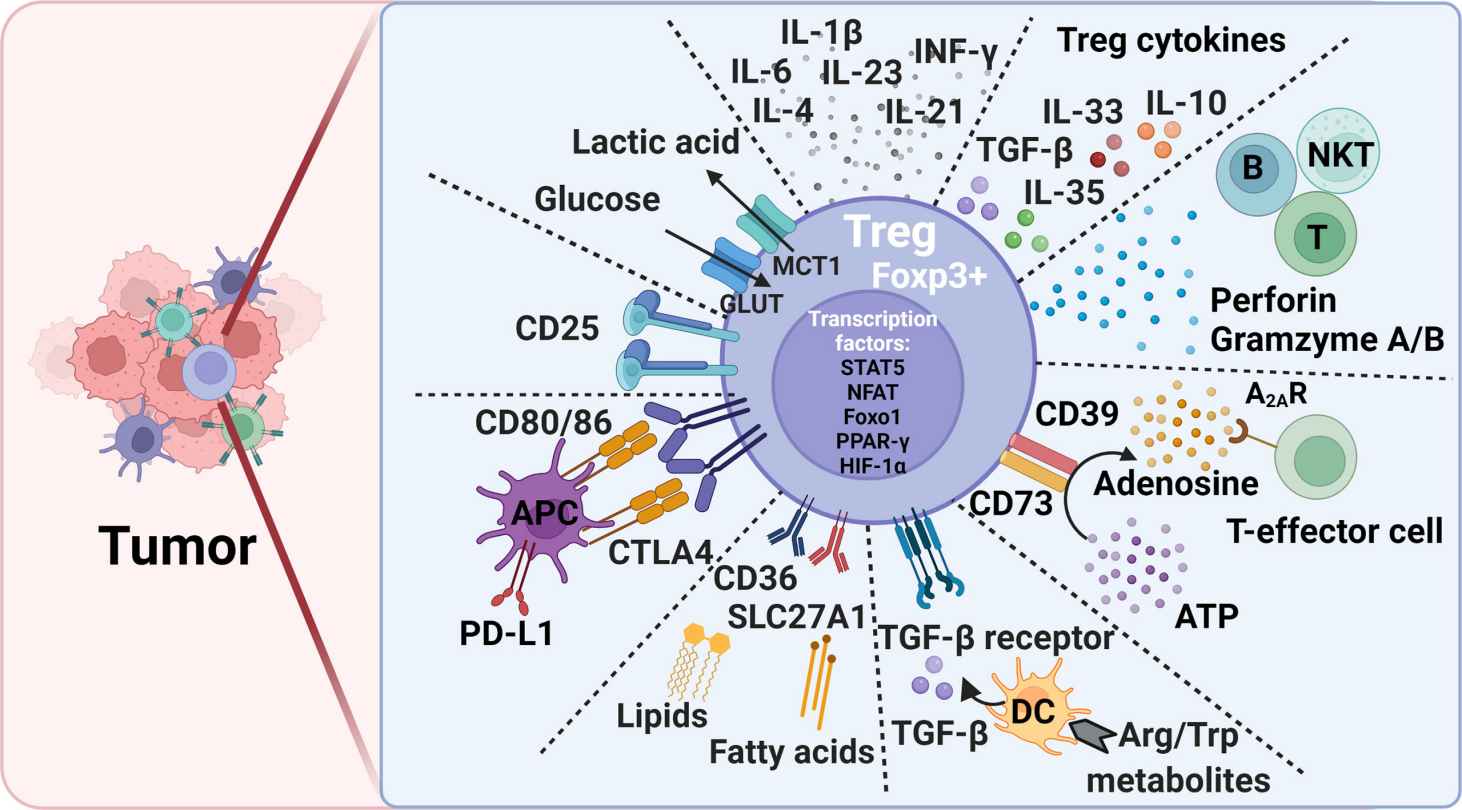
Treg-mediated immunosuppression mechanisms and potential therapeutic targets to alleviate immunosuppression in tumors. Foxp3, forkhead box P3; CTLA4, cytotoxic T lymphocyte antigen-4; PD-L1, programmed death ligand 1; DC, dendritic cell; Trp, L-tryptophan; Arg, L-arginine; ATP, adenosine triphosphate; Glut, Glucose transporters; MCT1, monocarboxylate transporter 1; STAT5, signal transducer and activator of transcription 5; NFAT, Nuclear factor of activated T cells; PPAR-γ, peroxisome proliferator- activated receptor-γ; HIF-1α, hypoxia-inducible factor 1α; Foxo1, Forkhead Box O1.

Treg surface molecules are among the essential Treg cell intrinsic molecules that respond to the tissue environment and alter Treg stability, plasticity, and tissue-specific heterogeneity. Common surface regulatory molecules include CD25, CTLA-4, TGF-β receptor, CD36, SLC27A1, and glucocorticoid induced tumor necrosis factor receptor (GITR). Expression of these molecules is required for Treg survival and immunosuppression. Foxp3 transfection of CD4^+^CD25^-^ primitive T cells transformed these cells into CD4^+^CD25^+^ Tregs cells that expressed these surface markers (39–41). Tregs regulate T-cell immune response using surface CTLA-4. CTLA-4 or even its truncated form without the cytoplasmic portion regulates the expression of CD80/86 on the surface of APCs during Treg-APC conjugation and immune synapse formation. This process allows CLTA-4 to remove CD80/86 on APC surface *via* a trogocytosis mechanism, followed by trans-endocytosis for intracellular degradation (40, 42, 43). Decreased CD80/86 expression on APC surface reduces CD28-mediated T-cell stimulation due to cell-extrinsic ligand depletion (40), causes CD80/programed death ligand-1 (PD-L1) heterodimer disruption, and increases free PD-L1 levels from the APCs (43) (**Figure 1**). Tregs in VAT express CD36 to uptake long-chain fatty acid and contribute to lipid accumulation in obese subjects (44). Short-chain fatty acids also promote iTreg differentiation (45, 46). In brain tumors, high percentages of Tregs express CD36 and SLC27A1 (fatty acid transporters). Inhibition of lipid uptake with sulfo-N-succinimidyl oleate (SSO) or fatty acid oxidation (FAO) with etomoxir prevented Treg immunosuppressive activity under this environment (47). Intratumoral Tregs showed over 15-fold increase of CD36 expression compared with those in the lymphoid organs. CD36 expression supports the suppressive activity of intratumoral Tregs. Disruption of CD36 selectively stimulated intratumoral Treg apoptosis and impaired tumor Treg accumulation and suppressive activity. Therefore, selective depletion of CD36 in Tregs suppressed tumor growth, decreased intratumoral Treg cells, and enhanced the anti-tumor activity in tumor-infiltrating lymphocytes without distrusting the immune homeostasis (48).

### Secreted Cytokines

Tregs also secrete suppressor cytokines, including TGF-β, IL-10, IL-35, and IL-33 (**Figure 1**). TGF-β and IL-10 inhibit the function of DCs and CD8^+^ T-effector cells and promote the transformation of CD4^+^ T cells into Tregs. TGF-β plays an essential role in Treg differentiation and controls the Treg suppressive activity (42). TGF-β signaling positively regulates Treg-dependent and -independent mechanisms of T-cell development and homeostasis (49). TGF-β signaling is also essential for Treg survival (50). When IL-10 induces M2 macrophages polarization to promote tumor immune escape during tumor progression (51, 52), IL-35 secreted from Tregs induces non-Tregs that inhibit other cells by the “infectious tolerance” mechanism (53, 54). Treg development, maintenance, accumulation, and immnosupression function in VAT and tumor tissues depend upon the expression of TCR, Foxp3, and IL-33. In tumor tissues, IL-33 deficiency attenuated Treg immunosuppressive activity against tumor growth. This activity of IL-33 was IL-33 receptor ST2-independent but depended on the NF-κB-T-bet-dependent IFN-γ production (55). In contrast, Treg development and maintenance in VAT was dependent upon the high expression of ST2 (56). IL-33 treatment increased ST2^+^ Tregs in VAT in obese mice (57). TCR : MHC-II interactions are also required for VAT Treg development and maintenance (58). In contrast, many extrinsic cytokines play different roles in Treg immunosuppression (**Figure 1**). Tregs lost their stability or proliferation when cells were exposed to inflammatory cytokines IL-6 and leptin and even IL-4 (19, 59, 60). Tregs became Foxp3^-^ cells that express inflammatory cytokines and failed to mediate immune suppression (61–63). Tregs also express leptin receptor, and leptin decreases Treg proliferation. Leptin is an important environment cue in the adipose tissue to modulate Tregs (60). CD44^hi^ memory T cells release IL-4, IL-21, and IFN-γ that inhibit TGF-β-induced Foxp3 expression (64). IL-6 and downstream STAT3 activation, as well as IL-23, IL-1β, and IL-21 are critical for Treg-to-Th17 conversion (65–67).

### Transcription Factors

In the nucleus, besides Foxp3, other transcription factors, such as STAT5, NFAT, Foxo1 (Forkhead Box O1), PPAR-γ (peroxisome proliferator- activated receptor-γ), and HIF-1 (hypoxia-inducible factor 1) control Treg development and maintenance by binding to the Foxp3 gene promoter and regulating Foxp3 gene expression (44, 68–71) (**Figure 1**). Foxo1 deficiency curtailed Treg development. Tregs from Foxo1-deficient mice were non-functional, did not express CTLA-4, and TGF-β failed to induce Foxp3 expression or to diminish T-bet expression (70). The expression of PPAR-γ is critical for establishing Treg transcriptional program and homeostasis, although this function of PPAR-γ seems mainly in VAT but less so in other organs (44). Hypoxia increases the expression of HIF-1α as a negative regulator of iTreg differentiation that promotes Th17 differentiation (72, 73).

### Amino Acid and Nucleic Acid Metabolites

Tumor cells modulate several environmental cues to affect tumor-resident Treg generation and function. Different from lymphoid tissue Tregs, those in tumors are often in activated state with a metabolic signature. Subtle perturbations in metabolic signaling impact tumor-resident Treg cell homeostasis and function (33). Basal amino acid catabolism maintains immune homeostasis, but increased amino acid catabolism enhances immune suppression. L-tryptophan (Trp) and L-arginine (Arg) are probably the most important immune response regulatory amino acids. While TDO (2,3-dioxygenase), IDO-1 and IDO-2 catabolize Trp, iNOS (inducible nitric oxide synthetase), and arginases Arg1 and Arg2 catabolize Arg. Reduction in Treg numbers is associated with reduced Trp and Arg catabolism. DCs express IDO to induce Foxp3^+^ Treg generation by inhibiting DC IL-6 production (74, 75). In mice, administration of Trp metabolite 3-hydroxyanthranthranilic acid (3-HAA) enhanced TGF-β secretion in DCs, increased Treg cells, and reduced Th1 and Th17 cell conversions (76, 77) (**Figure 1**). IDO has brought to the attention due to its broad expression in a variety of human tumor types. Under the tumor microenvironment, the immunosuppressive Tregs express lipid phosphatase PTEN (phosphatase and tensin homolog). IDO activates PTEN as a mechanism to maintain Treg immunosuppressive activity (78). Active IDO also maintains Treg immunosuppressive activity by suppressing IL-6 expression from DCs and blocking Treg to Th17 cell conversion. In a mouse melanoma model, IDO inhibitor together with anti-tumor vaccine increased DC IL-6 secretion, Treg to Th17 conversion, and CD8^+^ T-cell activity and anti-tumor efficacy (74). In addition, suppression of Arg1 and Arg2 activities inhibited Treg proliferation and promoted tumor antigen-specific T-cell tolerance. High levels of Foxp3^+^ Tregs in the tumor environment and the absence of Arg2 significantly impacted the survival of patients with head and neck squamous cell carcinoma (79, 80).

Extracellular purine metabolites regulate Foxp3 expression *via* the pro-inflammatory adenosine triphosphate (ATP) and anti-inflammatory adenosine. Tregs are more sensitive to oxidative stress than T-effector cells due to the low level of NRF2 (nuclear factor-erythroid factor 2-related factor 2, a key transcription factor for antioxidant responses) in Tregs. Oxidative stress induces Treg apoptosis, followed by ATP release. Apoptotic Tregs express the ectonucleotidases CD39 and CD73 that convert extracellular ATP into immunosuppressive adenosine to increase Treg suppressive function (53, 81, 82). Adenosine binds to the A_2A_ receptor (A_2A_R) to inhibit T-effector cell activity (83) (**Figure 1**). Activation of the adenosine signaling pathway can lead to enhanced Treg function, impaired APC function, and inhibition of NK cell activation (84). While apoptotic Tregs suppressed T-cell activation and tumor necrosis factor-α (TNF-α) and IL-2 expression, pharmacological inhibition of A_2A_ inhibitor blocked these Treg activities (81).

### Glucose Uptake and Glycolysis

Tregs use mitochondrial metabolism and oxidative phosphorylation for energy production (85). Treg cell extrinsic nutrients regulate oxidative phosphorylation. High glucose conditions, glucose transporter-mediated glucose uptake, or glucose avidity in Tregs correlate with Treg poor suppressive function and instability (86). In contrast, glucose deprivation drives Foxp3 expression, and shifts the T-cell differentiation from Tconv to iTregs (72, 85, 87). Intratumoral Tregs metabolize the glycolytic by-product lactic acid to support their proliferation and suppressor function. Deletion of the lactate transporter monocarboxylate transporter 1 (MCT1) in Tregs slowed tumor growth and increased responses to immunotherapy (86) (**Figure 1**). Together, elevated glycolysis can be detrimental to Treg. Accelerated glycolysis in tumor cells enhances glucose consumption and leads to increase of fatty acids. The metabolism of fatty acid then promotes Treg development (88). Increased glycolysis also promotes Th1 cell differentiation by epigenic regulation of the *Ifng* gene locus (89). Inhibition of glycolysis promotes the induction of Foxp3 expression in response to TGF-β and IL-2 stimulations (72, 90). Glycolytic enzyme enolase-1 also binds to the Foxp3 promoter and represses the expression of a transcription isoform of Foxp3 that is important for Treg suppressive activity (91). T cells use glycolysis during T-effector cell differentiation. While programmed cell death 1 (PD-1) ligation leads to defective T-cell glycolysis, amino acid metabolites and CTLA-4 inhibit T-cell glycolysis (92), supporting a mechanism by which Tregs control T-cell glycolysis and T-effector cell differentiation. Inhibition of the glycolytic pathway with the glucose analogue 2-deoxyglucose blocked Th17 development while promoting Treg generation (72).

The regulation of tissue-specific Treg differentiation, maintenance, and immunosuppression function has been summarized elsewhere (33). In addition to what we discussed above, many other metabolites also affect Treg pathobiology. For example, Vitamin A metabolite all-*trans* retinoic acid (RA) produced by DC subsets promote Foxp3 expression (93–96). The ten-eleven translocation (TET) family enzymes activate TSDR demethylation (97–99). CNS2 is demethylated in a TET-dependent manner in Foxp3^+^ iTregs (98). Vitamin C shows its activity to induce TET enzymatic activity in iTregs, thereby providing a new mechanism to stabilize iTregs (98, 100, 101). Tregs also suppress the activities of B cells, natural killer T cells (NKT), and cytotoxic T lymphocyte by secreting perforin and granzymes A and B (102, 103) (**Figure 1**). Tregs also inhibit the activation of type II innate lymphocytes, including NKT cells, mast cells, basophils, and eosinophils (104).

## Regulatory T-Cell Targeting Therapies

### Anti-CTLA-4 Monoclonal Antibody

CTLA-4 is an inhibitory receptor on Treg surface and is the most studied and widely used drug target in the clinic. Although Tregs inhibit the immune function *in vivo* through a variety of ways, deficiency of the CTLA-4 pathway in Tregs makes it difficult for Tregs to maintain their self-tolerance and immune homeostasis even if other inhibitory mechanisms are more active to compensate for the defects. CTLA-4 is an important negative regulator of T-cell responses and a key molecular target for Treg inhibition in physiological and pathological immune responses, including autoimmunity, allergy, and tumor immunity (105). The binding of CTLA-4 with CD80/86 molecule cuts off the CD80/86-CD28 pathway, an important step of T-cell activation. If the CD80/86-CD28 pathway is blocked, T cells become inactive in immune response. It is known that radiotherapy upregulates the expression of CD80 on APC surface. CTLA-4 on Treg surface has higher affinity to CD80, which leads to enhanced immunosuppression after radiotherapy. Therefore, the anti-CTLA-4 antibody therapy can be carried out at the same time of radiotherapy to achieve better anti-tumor immune effect. At present, the anti-CTLA-4 antibody drug mainly refers to iplimumab (IPI). It is the first Treg-targeted drug approved for clinical use, as the first choice for the treatment of advanced malignant melanoma. To enhance the CTLA-4 single drug therapy efficacy, Rudqvist et al. tested and found that combination of radiotherapy and anti-CTLA-4 antibody drug significantly increased the number of infiltrating lymphocytes and improved the survival rate in a mouse breast cancer model (106). Similarly, A phase II clinical trial evaluated the efficacy of local radiotherapy and IPI in patients with metastatic non-small cell lung cancer. The results showed that the objective response rate (ORR) and progression free survival (PFS) were all significantly better in patients received the combined therapy than those from the IPI therapy alone (107). Consistently, in a mouse liver metastasis model, combination of radiotherapy and anti-CD25/CTLA-4 antibody therapy increased tumor CD8^+^ T-cell accumulation with concurrent decrease of tumor Tregs, suppressed locally irradiated and abscopal unirradiated tumor growth, and improved overall survival rate. Therefore, combined radiotherapy with anti-CTLA-4 antibody reduced liver metastasis (108). Yet, not all studies yielded the same conclusion. A retrospective analysis of 133 tumor samples from patients with metastatic non-small cell lung cancer, melanoma, or renal cell cancer after receiving radiotherapy with or without combined immunotherapy showed that the combination of focal palliative radiation and CTLA-4 and/or PD-1 inhibitors was well tolerated. Patients received the combination therapy experienced more immune-related adverse events than those received either therapy individually (107). These observations might be due to the small patient sample size and many different types of treatments and combinations.

### Anti-GITR Monoclonal Antibody

GITR is a member of the TNF receptor protein family. In recent years, GITR has been widely studied as a promising Treg target. Studies have shown that the interaction between GITR and GITR ligand provides costimulatory signals for CD4^+^ and CD8^+^ T cells, activates T-effector cells, and suppresses Treg inhibitory activity (109). An agonistic anti-GITR monoclonal antibody or GITR ligands to activate GITR signaling can inhibit CD4^+^Foxp3^+^ Treg immunosuppressive activity and induce T-effector cell resistance to CD4^+^Foxp3^+^ Treg-mediated immunosuppression (110). The agonist GITR monoclonal antibody enhanced the anti-tumor response by increasing the activity of T-effector cells and reducing the invasion of Tregs (111). TRX-518, a GITR monoclonal antibody, was applied to 40 patients with metastatic solid tumors such as melanoma, non-small cell lung, and colorectal cancers in a phase I study. Single dose of TRX518 up to 8 mg/kg was well tolerated (NCT01239134). A phase I study of AMG228, another GITR agonist, exhibited favorable pharmacokinetics in patients with advanced solid tumors, but there was no evidence of T-cell activation or anti-tumor activity with AMG 228 monotherapy (NCT02437916) (112). In a mouse model of glioma, anti-GITR monoclonal antibody therapy combined with the radiotherapy increased the ratio of CD4^+^ T cells to Tregs, promoted tumor regression, and significantly improved mouse survival rate (113). Therefore, the effect of GITR on radio-sensitivity is worth to explore further in human trials.

### Anti-CD25 Antibody

CD25 antigen, as IL-2 receptor, is a 55-kDa single chain glycoprotein, and is mainly expressed by activated T cells. IL-2 is necessary for the expansion of CD8^+^ cytotoxic T lymphocyte (CTL) cells. In tumor microenvironment, Tregs express IL-2 receptors with high affinities that surpass the T-effector cells to obtain limited IL-2. Therefore, Tregs gain greater proliferation advantage than the T-effector cells do to promote immunosuppression (114, 115). Oweida et al. showed that anti-CD25 antibody treatment combined with the radiotherapy enhanced the T-cell cytotoxicity and induced the tumor antigen-specific memory response, which cured 57.1% of mouse tumors (116). To confirm further the effect benefit of this combination therapy, Ji et al. proved that radiotherapy combined with anti-CD25 monoclonal antibody therapy reversed the increase of PD-1 expression on CD8^+^ T cells and CD4^+^ T cells during the radiotherapy, thereby inhibiting the tumor growth and improving the overall survival rate (108). Yet, few similar studies failed to obtain clinically significant enhancement of Treg-depletion-associated beneficial effects (117–119). One possible reason is that the activated T-effector cells also express CD25. Anti-CD25 antibody treatment may also reduce the activity of activated T-effector cells, thereby weakening the anti-tumor immunity that was enhanced by Treg depletion. Anti-CD25 antibody therapy has also been evaluated in clinical trials. To breast cancer patients, use of an anti-CD25-depleting antibody daclizumab stabilized cancer progression and increased overall survival time in a median follow-up of 22.3 months (120). Meanwhile, the administration time and dose of monoclonal antibody may also be important factors to affect the tumor immunity between Tregs and T-effector cells.

### IDO-1 Inhibitor

IDO-1 is a naturally occurred immune regulatory enzyme. Prior studies have found many potential immunosuppressive mechanisms of IDO-1, among which the main mechanism is Trp catabolism in the microenvironment. The relationship between tumor growth and the increase of Trp catabolism becomes more and more recognized. Trp is metabolized into kynurenine by the IDO limiting enzyme, which blocks or inactivates the cell cycle of T-effector cells. This progress plays a direct role in tumor immune escape to promote Treg maturation and activation. IDO inhibitors have been intensively studied in recent years. The radiotherapy could alter IDO-mediated immune activity, and there was a strong correlation between the IDO activity and survival outcomes in patients under a radiotherapy (121). Therefore, the use of IDO inhibitors during radiotherapy can delay the tumor growth. IDO inhibitors in combination with radiotherapy down-regulated the number of CD4^+^CD25^+^Foxp3^+^ Tregs and DC expression of MHC-II in the spleen, along with decreased expression of inhibitory receptors (PD-1 and T cell immunoglobulin domain and mucin domain 3) and ligands (galetin-9 and B- and T-lymphocyte attenuator) to prevent T-cell exhaustion, while DCs and T-effector cells were activated (122). This combination therapy enhanced anti-tumor immunity and inhibited tumor progression.

### Immunosuppressive Cytokines

Other therapy related to the inhibition of Treg function is to target the Treg immunosuppressive cytokines, such as TGF-β and IL-10. Hou et al. showed that TGF-β chimeric antigen receptor (CAR) T cells promoted the anti-tumor immune response by alleviating TGF-β-mediated immunosuppression, and reduced Foxp3^+^ Treg differentiation (123). IL-10 deficiency diminished the Treg-mediated immunosuppression by suppressing neuropillin expression in Treg cells in mouse tumor models (124). Furthermore, pegylated-recombinant human IL-10 has been used in phase I clinical trial (NCT02009449) to decrease TGF-β expression and up-regulate serum levels of IFN-γ and IL-18 in patients with tumors (125). Pegylated-recombinant human IL-10 has also been used to test its efficacy in patients with metastatic pancreatic cancer (phase III clinical trial, NCT02923921) (126).

### Dual Therapy

**Figure 2:**
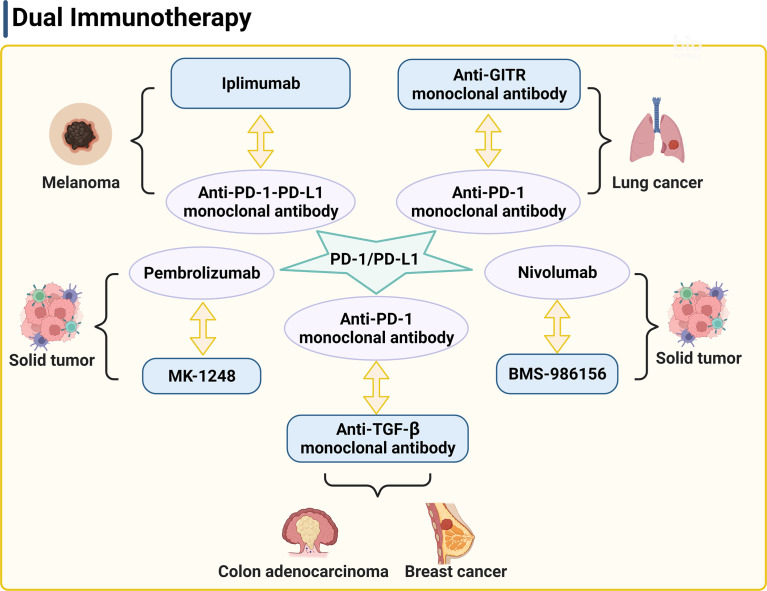
PD-1/PD-L1 is a key target of the dual therapy. The strategy of dual therapy contains anti-PD-1/PD-L1 antibodies to enhance the anti-tumor responses. PD-1, programmed cell death 1; PD-L1, programmed death ligand 1; GITR, glucocorticoid induced tumor necrosis factor receptor.

The combination of immunotherapy and radiotherapy enhances the anti-tumor immune response through various mechanisms of immune escape, thereby improving the survival rate more than any single therapy. PD-1 is a type I transmembrane glycoprotein, which was first discovered in 1992 (127). It is mainly expressed on the surface of activated T cells, but its ligand PD-L1 is expressed in many cell types, such as APCs, macrophages, and tumor cells, etc. Under normal circumstances, PD-1 binding of PD-L1 can stop the continuous activation of T cells and prevent the occurrence of autoimmune diseases. Under pathological conditions however, PD-L1 expression from tumor cells was significantly higher than that in other normal cells. The interaction of PD-1 and PD-L1 inhibits the activation of anti-tumor T cells, thereby inducing tumor growth. Many studies have shown that the radiotherapy combined with the anti-PD-1-PD-L1 therapy dramatically suppressed tumor growth. For example, combined use of irradiation and anti-PD-L1 antibody therapy reduced the local accumulation of tumor-infiltrating myeloid-derived suppressor cells with feature of immune suppression (128), which altered the tumor immune microenvironment (129), supporting a close interaction between irradiation, T cells, and the PD-L1/PD-1 axis. Pembrolizumab is a highly selective humanized PD-1 monoclonal antibody. A randomized phase II clinical trial (NCT02492568) of 92 patients of advanced non-small cell lung cancer showed that combined therapy of pembrolizumab after radiotherapy showed much higher overall response rate, progression-free survival rate, and overall survival time than those in patients received pembrolizumab alone (130). A retrospective analysis of 133 patients with metastatic non-small cell lung cancer, melanoma, or renal cell cancer after receiving radiotherapy with or without combined immunotherapy with CTLA-4 and/or PD-1 inhibitors showed that patients were well tolerated with the combination of focal palliative radiation and CTLA-4 and/or PD-1 inhibitors (107). Twyman-Saint Victor et al. put forward the hypothesis that the optimal response in melanoma and other cancer types required radiotherapy together with the anti-CTLA-4 and anti-PD-L1/PD-1 antibodies (**Figure 2**) (131). They demonstrated that anti-CTLA-4 monoclonal antibody IPI combined with radiotherapy made the tumor substantially subsided in both clinical trials and mouse models of metastatic melanoma, although drug resistance can be a potential concern. Of note, radiotherapy, anti-CTLA-4 antibody, and anti-PD-L1/PD-1 antibody promote tumor immune response with distinct mechanisms. Radiotherapy enhances intratumoral T-cell TCR repertoire diversity, anti-CTLA-4 antibody inhibits Treg and increases CD8^+^ T cell to Treg ratio, and anti-PD-L1/PD-1 antibody reverses T-cell exhaustion and increases T-cell expansion. Melanoma patients or mice with high expression of PD-L1 on melanoma cells failed to respond to radiotherapy or combined therapy and these patients showed much shorter progression-free survival and overall survival. High expression of PD-L1 on melanoma cells allowed tumors to escape the anti-CTLA-4-based therapy (131). Therefore, the known drug resistance may rely on the PD-L1 upregulation on melanoma cells, leading to T-cell depletion. The results from this clinical study suggest the importance of combined application of anti-CTLA-4 monoclonal antibody, anti-PD-1-PD-L1 monoclonal antibody, and radiotherapy, which activate the anti-tumor responses in multiple mechanisms.

In a mouse model of anti-PD1 antibody therapy-resistant non-small cell lung adenocarcinoma, combination of anti-GITR monoclonal antibody therapy, anti-PD-1 monoclonal antibody therapy, and radiotherapy also significantly increased the numbers of CD4^+^ and CD8^+^ effector memory cells in blood, spleen, and tumor draining lymph node, enhanced tumor-specific immune response, and improved the survival rate (**Figure 2**) (132). Half of the mice showed no tumors. Furthermore, an anti-GITR antibody (BMS-986156) combined with a PD-1 inhibitor (nivolumab) was well tolerated with low immunogenicity in patients with advanced solid tumors (**Figure 2**). Antitumor activity was observed with combined use of BMS-986156 and nivolumab at doses predicted to be biologically active (NCT02598960). In a trial of solid tumors, including colorectal cancer, melanoma, and renal cell carcinoma, 17 patients who received a combination therapy of an anti-GITR antibody agonist (MK-1248) and humanized PD-1 monoclonal antibody pembrolizumab reached to an objective response rate of 18% versus no response in 20 patients who received MK-1248 monotherapy (**Figure 2**) (133). In preclinical experiments, anti-TGF-β monoclonal antibody and anti-PD-1 monoclonal antibody combination with radiotherapy also showed effective antitumor activity (**Figure 2**) (134, 135). Together, these studies suggest the potential that dual therapy combined with or without radiotherapy overcomes common tumor resistance concerns.

## Conclusions

In this short review, we overviewed the origins, functions, and potential clinical applications of Tregs in cancer patients. Tregs participate in the process of immune dysfunction. Based on the mechanism of Tregs, some immunotherapies have demonstrated their clinical efficacy in tumor controls, and candidate drugs have proceeded to clinical trials (**Table 1**). In particular, the combination of immune inhibitory therapies and radiotherapy has demonstrated its success of inhibitory efficacies in tumor progression, and potential synergistic mechanisms of combination therapy may explain the positive results seen in both clinical trials and experimental models. There are accumulating evidences to support the hypothesis that radiotherapy combined with anti-Treg therapy reverses Treg immunosuppressive activity and enhances the efficacy of radiotherapy. Therefore, the revised Treg immunotherapy has become a potential breakthrough point of cancer therapy. It is necessary to explore more Treg functions and inhibitors in the future to effectively control or reverse the immunosuppressive activities of Tregs and to optimize cancer combinational therapy.

**Table 1:** Selected immunotherapy targets and clinical trials.

Target	Immunotherapy	Clinical trails
CTLA-4	Iplimumab (IPI)	NCT02221739
GITR	TRX-518	NCT01239134
	AMG228	NCT02437916
	BMS-986156	NCT02598960
	MK-1248	NCT02553499
CD25	Daclizumab	–
IDO-1	IDO inhibitors	–
Cytokines	Pegylated-recombinant human IL-10 1D11, a TGFβ-neutralizing antibody	NCT02009449; NCT02923921 -
PD-1	Pembrolizumab Nivolumab	NCT02492568 -

As we discussed above, there are many essential molecules that regulate Treg functions. These include Treg surface molecules, cytokines, transcription factors, amino acid and nucleic acid metabolites, and glucose and glycolytic metabolites. Targeting these molecules and their pathways has led to the development of varies immunotherapy approaches to target the immunosuppressive activity of Tregs against different types of tumors. Yet, while drug efficacy and target selection are of the primary interest of patients and physicians, the development of drug resistance has been the major hurdle in cancer therapy. Here we discussed the benefit of dual therapy by combining the radiotherapy with anti-CTLA-4 and/or anti-PD-1-PD-L1 antibody immunotherapy. While dual therapy may demonstrate synergistic beneficial effects against tumor progression, patients may still develop drug resistance. Immunosuppressive Tregs display adverse effects in tumor growth by down-regulating T-effector cell immune responses. Tregs play protective roles in cardiac, metabolic, autoimmune, and neurological diseases (136–139). We showed that adoptive transfer of Tregs in recipient mice blocked angiotensin II perfusion-induced abdominal aortic aneurysms (140), induced β3-adrenergic receptor agonist-induced adipose tissue thermogenic program (141), and blunted the development of spontaneous systemic lupus erythematosus (142). Therefore, one should consider these beneficial effects of Tregs before treating cancer patients, especially those with cardiovascular, metabolic, and neurological complications. Therefore, discovery of tumor-selective Treg functions and combination therapy to target multiple Treg immunosuppression pathways might be necessary. For example, CD36 expression in intratumoral Tregs was much higher than in those from lymphoid organs (48). CD36 targeting primes tumors to PD-1 blockade and elicited additive anti-tumor responses with the anti-PD1 antibody therapy (48). Tumor resident Tregs express high levels of unique signature genes *Ccr8, Tnfrsf8, Cxcr3*, and *Smasn1* (143, 144), which might serve as valuable targets for tumor immunotherapy. Further, non-lymphoid and tumor tissues differ in their metabolic environments, nutrient supplies, and intracellular metabolic requirements. Consideration of these factors may help develop new generations drugs for cancer patients by selectively targeting the immunosuppressive functions of intratumoral Tregs.

## Author Contributions

RG and JW drafted the manuscript. G-PS edited the manuscript. All authors contributed to the article and approved the submitted version.

## Funding

This work was supported by the Chinese Academy of Medical Sciences Innovation Fund for Medical Sciences (2021-I2M-1-016 to RG), National Natural Science Foundation of China (82100514 to RG), National Key Research and Development Program of China Grants (2019YFA0801804 and 2019YFA0801703 to JW), and grants from the National Heart, Lung, and Blood Institute (HL151627 and HL157073 to G-PS), and the National Institute of Neurological Disorders and Stroke (AG063839 to G-PS).

## Conflict of Interest

The authors declare that the research was conducted in the absence of any commercial or financial relationships that could be construed as a potential conflict of interest.

## Publisher’s Note

All claims expressed in this article are solely those of the authors and do not necessarily represent those of their affiliated organizations, or those of the publisher, the editors and the reviewers. Any product that may be evaluated in this article, or claim that may be made by its manufacturer, is not guaranteed or endorsed by the publisher.
